# A universal pipeline for mobile mRNA detection and insights into heterografting advantages under chilling stress

**DOI:** 10.1038/s41438-019-0236-1

**Published:** 2020-02-01

**Authors:** Ying Wang, Lingping Wang, Nailin Xing, Xiaohua Wu, Xinyi Wu, Baogen Wang, Zhongfu Lu, Pei Xu, Ye Tao, Guojing Li, Yuhong Wang

**Affiliations:** 1 Institute of Vegetables, Zhejiang Academy of Agricultural Sciences, Hangzhou, 310021 China; 2 Institute of Vegetables, Ningbo Academy of Agricultural Sciences, Ningbo, 315040 China; 3 State Key Laboratory for Quality and Safety of Agroproducts, Zhejiang Academy of Agricultural Sciences, Hangzhou, 310021 China; 4 Biozeron Biotechnology Co., Ltd., Shanghai, 201800 China; 5 Present Address: College of Life Sciences, China Jiliang University, Hangzhou, 310018 China

**Keywords:** Abiotic, Bioinformatics

## Abstract

Heterografting has long been used to enhance the chilling tolerance of temperature-sensitive crops, including watermelon, whose mechanism is known to involve bidirectional long-distance mRNA movements. Despite several studies reporting on mobile mRNA (mb-mRNA) profiles in plants, accurate identification of mb-mRNAs is challenging owing to an array of technical problems. Here, we developed a bioinformatical pipeline that took most of the known technical concerns into consideration and is considered to be a universal tool for mb-mRNA detection in heterografts. By applying this pipeline to a commercial watermelon–bottle gourd heterografting system, we detected 130 and 1144 mb-mRNAs upwardly and 167 and 1051 mb-mRNAs downwardly transmitted under normal and chilling-stress conditions, respectively. Quantitative real-time PCR indicated a high accuracy rate (88.2%) of mb-mRNA prediction with our pipeline. We further revealed that the mobility of mRNAs was not associated with their abundance. Functional annotation and classification implied that scions may convey the stress signal to the rootstock, subsequently triggering energy metabolism reprogramming and abscisic acid-mediated stress responses by upward movement of effective mRNAs, ultimately leading to enhanced chilling tolerance. This study provides a universal tool for mb-mRNA detection in plant heterografting systems and novel insights into heterografting advantages under chilling stress.

## Introduction

Watermelon (*Citrullus lanatus* L.), a member of the Cucurbitaceae family, is one of the top five most-consumed fresh fruits globally. In many areas of the world, chilling stress owing to early-spring low temperature is a major environmental stress threatening the watermelon industry^1,2^. Among the several techniques that are commonly used to cope with chilling stress, grafting is one of the most-effective and cost-saving approaches^3–5^. The bottle gourd (*Lagenaria siceraria* (Mol.) Standl.) is routinely used as a rootstock for grafting with watermelon because of its climate resiliency and high compatibility with watermelon^3,6,7^.

Recent studies have investigated the molecular mechanisms of watermelon–bottle gourd heterografting advantages from various perspectives. Liu et al.^8^ reported that 787 genes were differentially expressed between self-grafted watermelon and watermelon grafted onto bottle gourd. Wang et al.^9^ revealed that miRNAs whose target genes were involved in biological processes, such as polyamine biosynthesis and protein phosphorylation, were differentially expressed under chilling stress. Whereas the long-distance movement of mRNAs between the scion and rootstock has been documented in some model and nonmodel plant heterografting systems, little is currently known about this phenomenon in the watermelon–bottle gourd heterografting system.

Technically, large-scale identification of mobile mRNAs (mb-mRNAs) between a scion and a rootstock is enabled by RNA sequencing. Thieme et al.^10^ reported 2006 genes producing mb-mRNAs bidirectionally in *Arabidopsis* Col-0 and Ped-0 interecotypic heterografts. A similar number (2679) was reported in a grapevine heterografting system between *Vitis girdiana* and *Vitis palmata*^11^. Some studies have focused only on unidirectional mRNA mobilization. For example, 1163 mRNAs were detected as moving from scion to rootstock in a *Nicotiana benthamiana*/tomato heterograft^12^; a much larger number (346) were detected as moving from rootstock to scion in a cucumber–watermelon heterograft^13^; strikingly, only 138 mRNAs were identified as mobile from rootstock to scion in an *Arabidopsis*/*N. benthamiana* heterograft^14^.

The drastic inconsistency in reported mb-mRNA numbers may reflect the intrinsic patterns of long-distance mRNA mobilization in different systems/conditions, but it could also stem from analytical procedures. In principle, methodologies for large-scale mb-mRNA detection rely on the detection of SNPs in mRNA sequences between the scion and rootstock^10,11^, and hence, the sequence divergence level between a scion and a rootstock has a large impact on the result. Moreover, as SNP detection requires that the mRNA reads are first aligned to a reference genome, false positives or negatives can arise during this step if the scion or rootstock genotype used in grafting (“experimental genotype” hereafter) is not identical to that used for reference genome assembly (“reference genotype” hereafter). Although selecting reference genotypes to make heterografts is a solution to avoid this technical issue, most grafting combinations in agriculture are based on particular landraces/varieties, and hence, universal solutions are required to enable accurate mb-mRNA detection.

In this study, we developed an improved bioinformatical pipeline for bidirectional mb-mRNA identification that accounts for the known major technical limitations. By applying this pipeline to a commercial watermelon–bottle gourd heterograft combination, we demonstrated its accuracy and robustness. Novel insights into heterografting advantages were gained by analyzing mb-mRNA profiles.

## Results

### Heterografting conferred chilling tolerance to the scion

We used watermelon variety “8424” as the scion and bottle gourd variety “YongZhen” (hereafter “YZ”) as the rootstock to generate three graft combinations (8424/YZ = heterografts; 8424/8424 = 8424 homografts; YZ/YZ = YZ homografts).

**Fig. 1:**
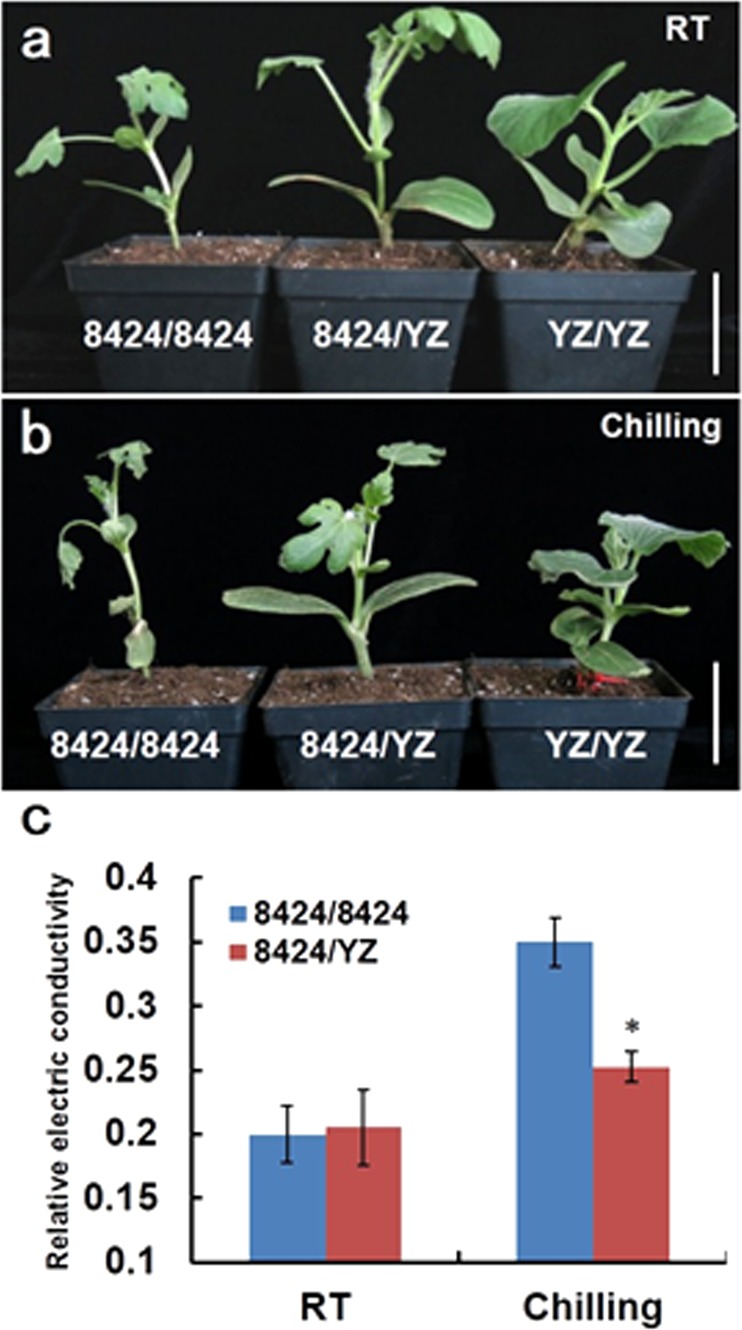
Phenotypic comparison of 8424/8424, 8424/YZ, and YZ/YZ seedlings. **a** Comparison of 8424/8424, 8424/YZ, and YZ/YZ seedlings under nonstress (RT) conditions; **b** comparison of 8424/8424, 8424/YZ, and YZ/YZ seedlings after chilling stress for 2 days; **c** relative electric conductivity (REC) of 8424/YZ and 8424/8424 seedlings under nonstress (RT) and chilling-stress (Chilling) conditions, respectively. Bars, 5 cm in **a** and **b**. Error bars are standard deviations of three replicates (Student’s *t* test: *P < 0.05).

The three graft combinations were compared for their performances under room temperature and chilling stress (Fig. 1a, b). After 2 days of chilling stress, the YZ/YZ homograft and 8424/YZ heterograft both exhibited a visibly more tolerant phenotype than the 8424/8424 homograft, which included mitigated wilting and leaf curling (Fig. 1b). An assay of leaf relative electrical conductance (REC), a physiological parameter indicative of cellular damage under stress conditions, showed that the REC values were significantly smaller in 8424/YZ than in 8424/8424 grafts under the chilling-stress condition (Fig. 1c), suggesting more-effective cellular structure maintenance in the former. Under nonstress conditions, no visible differences in morphology or REC values were observed between the graft combinations (Fig. 1a, c). These results demonstrated that grafting with YZ as a rootstock conferred chilling tolerance to the 8424 scion.

## Reconstruction of reference genomes for the scion and rootstock

**Fig. 2:**
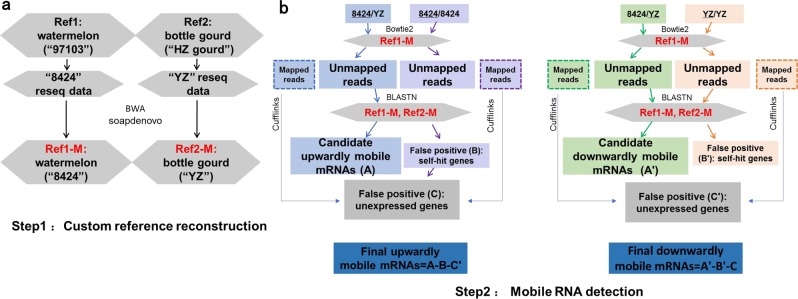
Bioinformatical pipeline for modified reference genome reconstruction and mobile mRNA detection. **a** Pipeline for modified reference genome reconstruction of watermelon (Ref1-M: watermelon-8424) and bottle gourd (Ref2-M: bottle gourd-YZ); **b** bioinformatic analysis workflow to identify the bidirectional mobility of mRNAs in watermelon–bottle gourd heterografts.

An important improvement in our bioinformatical pipeline is the inclusion of a step to correct for SNPs and indels between the experimental and reference genomes (Fig. 2; detailed information in materials and methods). Neither 8424 nor YZ had a publicly available reference genome. Therefore, an important step before in silico identification of mb-mRNAs was to modify the existing reference genomes by correcting for SNPs, indels, and genotype-specific sequences between the experimental and reference genotypes (Fig. 2a). Genome shotgun sequencing of 8424 and YZ generated 64,417,420 and 74,566,000 reads, with ~28.5× and 35.9× genome coverage, respectively (Table S1). A total of 43,966,737 and 49,830,767 paired-end reads from 8424 and YZ were mapped to the 97,103 (Ref1) and HZ gourd (Ref2) reference genomes with coverages of 79.58% and 77.53%, respectively (Table S1). The unmapped reads, which were owing to SNPs or structural variations between the experimental and reference genotypes, were used to modify the reference genomes (Ref1-M and Ref2-M). In total, 83,957 SNPs, 20,117 insertions and 15,036 deletions were incorporated to reconstruct the modified reference genome for 8424, and 310,921 SNPs, 50,899 insertions and 30,152 deletions were corrected for YZ. In addition to SNPs and indels, reads that were unique to 8424 or YZ were assembled into extra scaffolds (Table S2) to account for genotype-specific sequences.

### In silico identification of mb-mRNAs

**Fig. 3:**
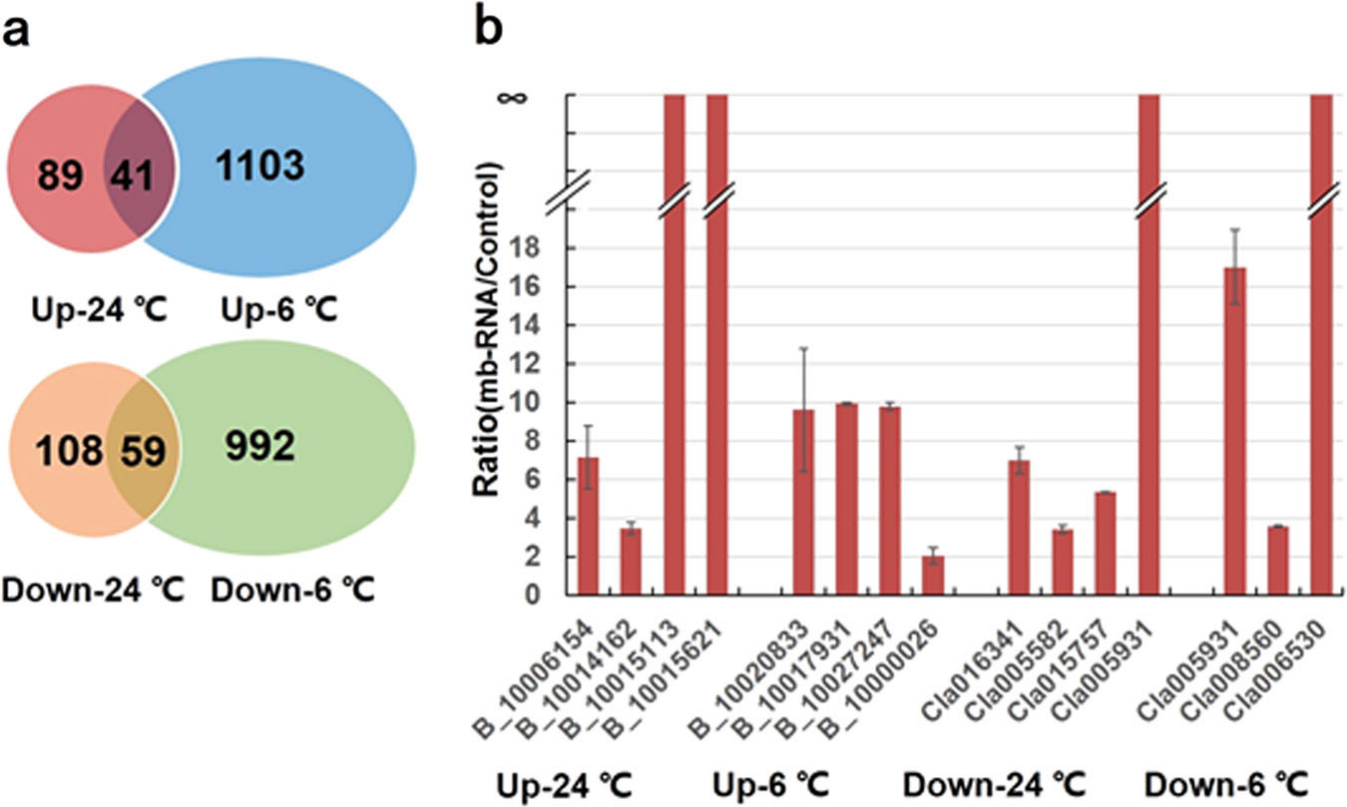
Venn diagram and qRT-PCR verification of mb-mRNAs in nonstress (24 °C) and chilling-stress (6 °C) conditions. **a** Venn diagram of upwardly and downwardly mb-mRNAs at 24 °C and 6 °C; **b** confirmation of the 15 investigated mb-mRNAs. For the bottle gourd gene codes, BG_GLEAN_ is abbreviated as B_ for clarity. The *y* axis indicates the ratio of gene expression level in the heterografts (for instance, the scion of the watermelon–bottle gourd heterograft) to that in the negative control (for instance, the watermelon homograft). Error bars are standard deviations of three replicates.

Next, a streamlined computational pipeline (Fig. 2b; detailed information in materials and methods) was applied to identify putative mobile mRNAs from the RNA-Seq data (Table S3). On average, we obtained 59.5 Gb to 73.1 Gb of sequence data for the heterografts under various conditions, which yielded more than eight times deeper coverage than regular RNA-Seq analysis. Here, the deep sequencing depth increased the likelihood of catching low-abundance mb-mRNAs, which are known to be common in plant heterografts^10,13^. The reads were first mapped to Ref1-M and Ref2-M by Bowtie, yielding an average of 11,203,470 unmapped reads from 8424/YZ to Ref1-M and 11,529,356.5 unmapped reads from 8424/YZ to Ref2-M in the two replicates. These reads were then BLASTn searched against the modified reference genomes, yielding 9,041,047.5 reads from 8424/YZ mapped to Ref2-M and 9,000,995.75 reads from 8424/YZ that mapped to Ref1-M. Notably, 41,511 reads (3.6% of the total unmapped reads) from the 8424/8424 homograft and 89,175 reads (7.0% of total unmapped paired reads) from the YZ/YZ homograft also mapped to Ref2-M and Ref1-M, respectively, suggesting considerable false-positive rates. After subtracting the false positives, a final set of 130 upwardly (from YZ to 8424) mb-mRNAs and 167 downwardly (from 8424 to YZ) mb-mRNAs were identified in the heterografts grown under normal conditions (Table S4). In contrast, 1144 upwardly mb-mRNAs and 1051 downwardly mb-mRNAs were identified from the heterografts grown under chilling stress (Table S4). Only 41 upwardly mb-mRNAs and 59 downwardly mb-mRNAs were shared between the stress and nonstress conditions (Fig. 3a). Combined, these results point to unexpectedly large effects of chilling stress on bidirectional mRNA mobilization (see Discussion).

### Validation of mb-mRNAs

To validate the results of the in silico prediction of mb-mRNAs, we randomly selected predicted mb-mRNAs (20 in total) from each of the four graft combinations for qRT-PCR analysis. Owing to the high level of sequence similarity between watermelon and bottle gourd, 3 of the 20 primer pairs failed to discriminate PCR products between the two species and were discarded from further analysis. According to the ratio of expression levels from the heterografts to the negative control (homografts), only 2 of the 17 predicted mb-mRNAs were judged as false positives in which no expression was detected in the sampled tissue of the heterograft or high expression was detected in the negative control. The remaining 15 predicted mb-mRNAs all showed much higher expression in the heterograft than in the negative control. For example, the ratio of the expression level of upwardly mb-mRNA (*BG_GLEAN_10015113*) between the watermelon–bottle gourd heterograft (scion part) and the negative control (watermelon homograft) was infinite. Therefore, an accuracy rate of ~88.2% (15/17) for the prediction of mb-mRNAs was obtained using our bioinformatical pipeline. Among the 15 verified mb-mRNAs that exhibited expected expression in leaves of the heterograft but no or weak expression in the control, four fell into the category of upwardly mb-mRNAs under normal growth conditions (up-24 °C), 4 into up-6 °C, 4 into down-24 °C and 3 into down-6 °C (Fig. 3b).

### mRNA mobility exhibited no association with mRNA abundance in source tissues

**Fig. 4:**
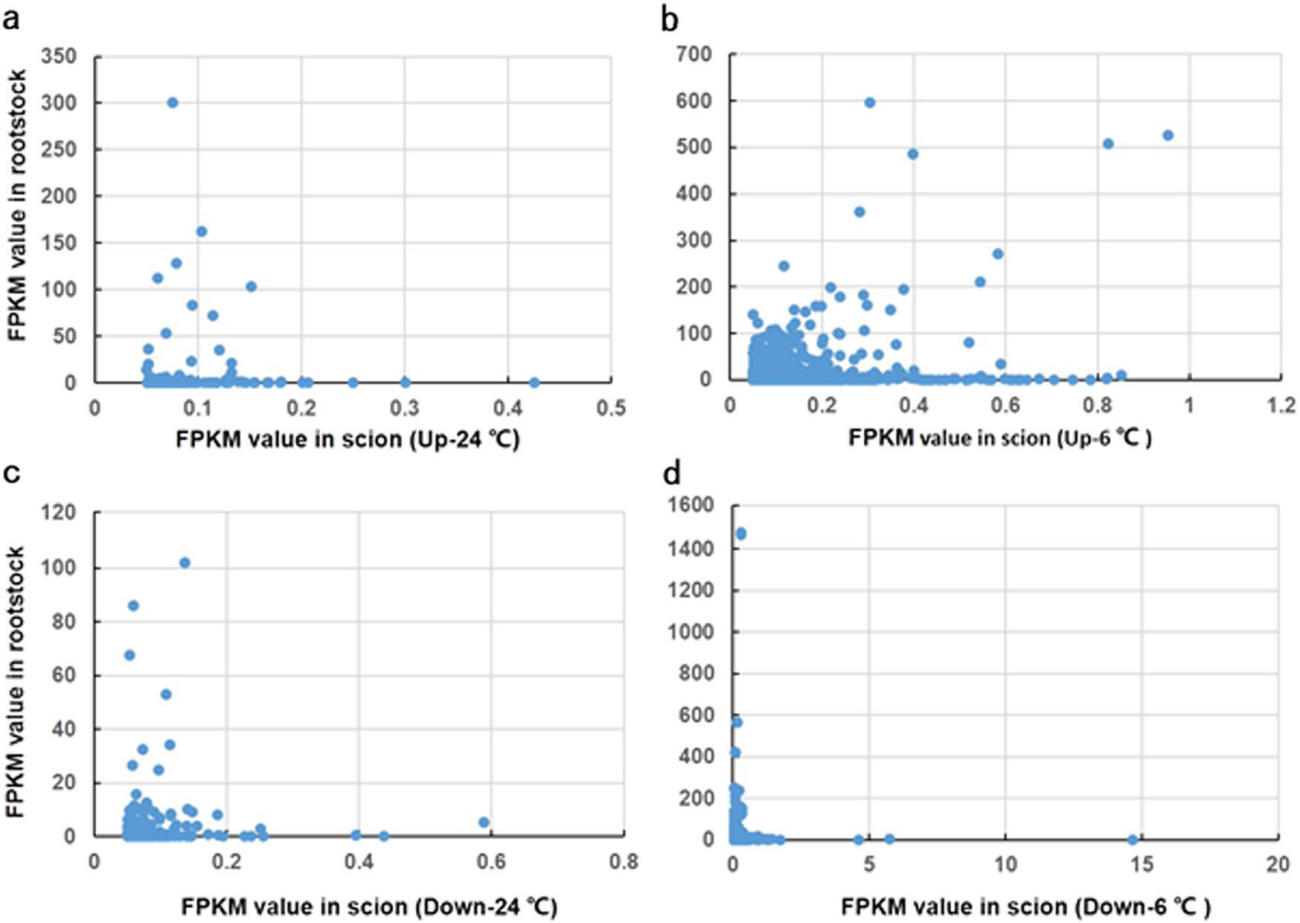
mb-mRNA transmission rates and their distribution patterns. **a** Plot of abundance for 130 upwardly mb-mRNAs at 24 °C in the scion and rootstock; **b** plot of abundance for 1144 upwardly mb-mRNAs at 6 °C in the scion and rootstock; **c** plot of abundance for 167 downwardly mb-mRNAs at 24 °C in the scion and rootstock; **d** plot of abundance for 1051 downwardly mb-mRNAs at 6 °C in the scion and rootstock.

**Fig. 5:**
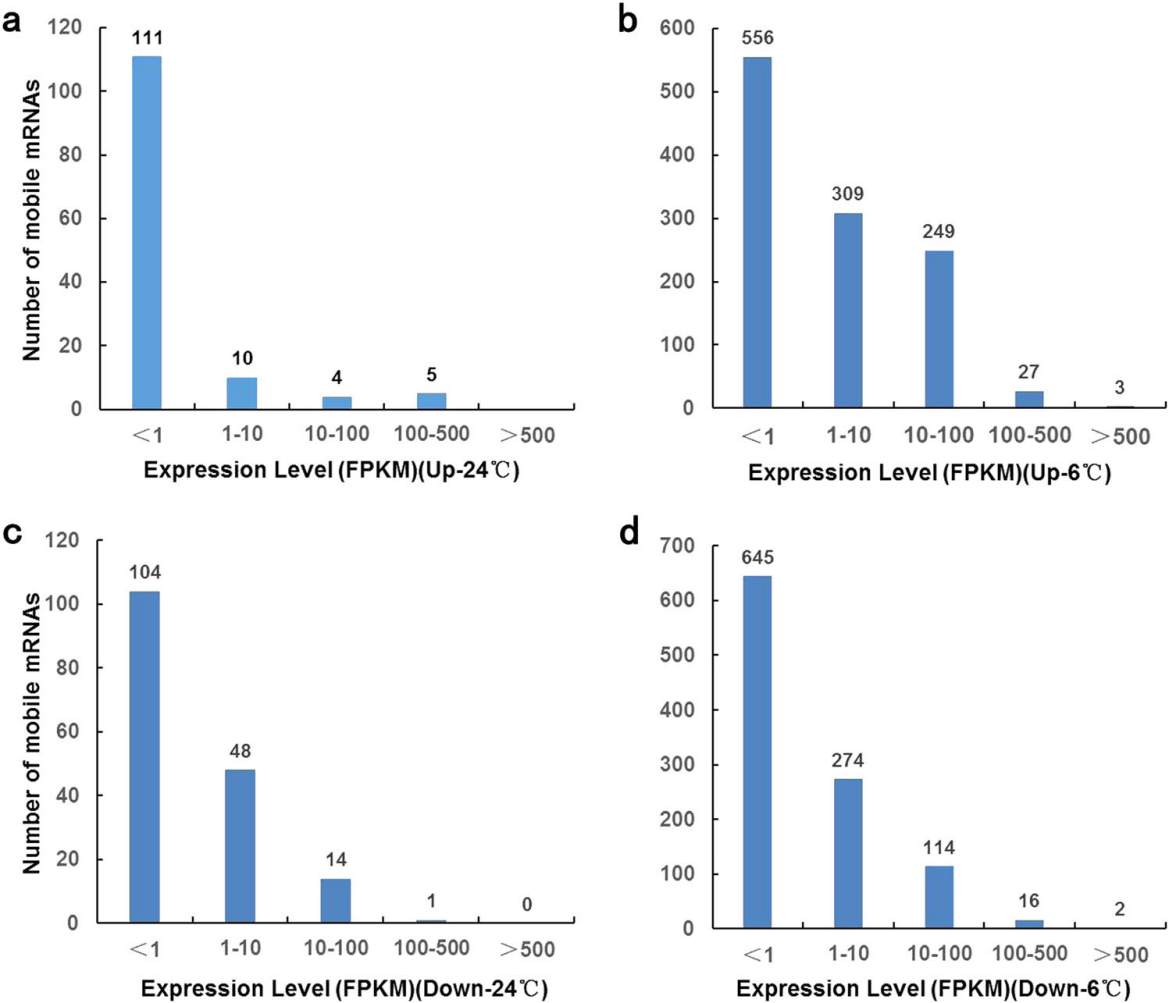
Distribution of the number of mb-mRNAs in different conditions. **a** Distribution of the 130 upwardly mb-mRNAs at 24 °C; **b** distribution of the 1144 upwardly mb-mRNAs at 6 °C; **c** distribution of the 167 downwardly mb-mRNAs at 24 °C; **d** distribution of the 1051 downwardly mb-mRNAs at 6 °C. The *x* axis indicates the FPKM value. The *y* axis indicates the number of mobile mRNAs. Numbers on the histograms indicate the exact numbers of mb-mRNAs.

Whether the long-distance mobility of mRNAs is associated with their cellular abundances is currently debated. To explore this question in our system, we compared the abundances of mb-mRNAs and their relative expression levels in the tissues of origin. As shown in Fig. 4, for both the stress and nonstress conditions and regardless of the direction of movement, no obvious association was observed between the abundance of mRNAs and their long-distance mobility. In the four libraries, 48.6% to 85.4% of the mb-mRNAs were found to originate from low-expressed genes with an FPKM (fragments per kb of transcript per million fragments mapped) < 1, further indicating that high expression level is not a prerequisite for mRNA mobilization (Fig. 5).

### Functional characterization and classification of the mb-mRNAs

**Fig. 6:**
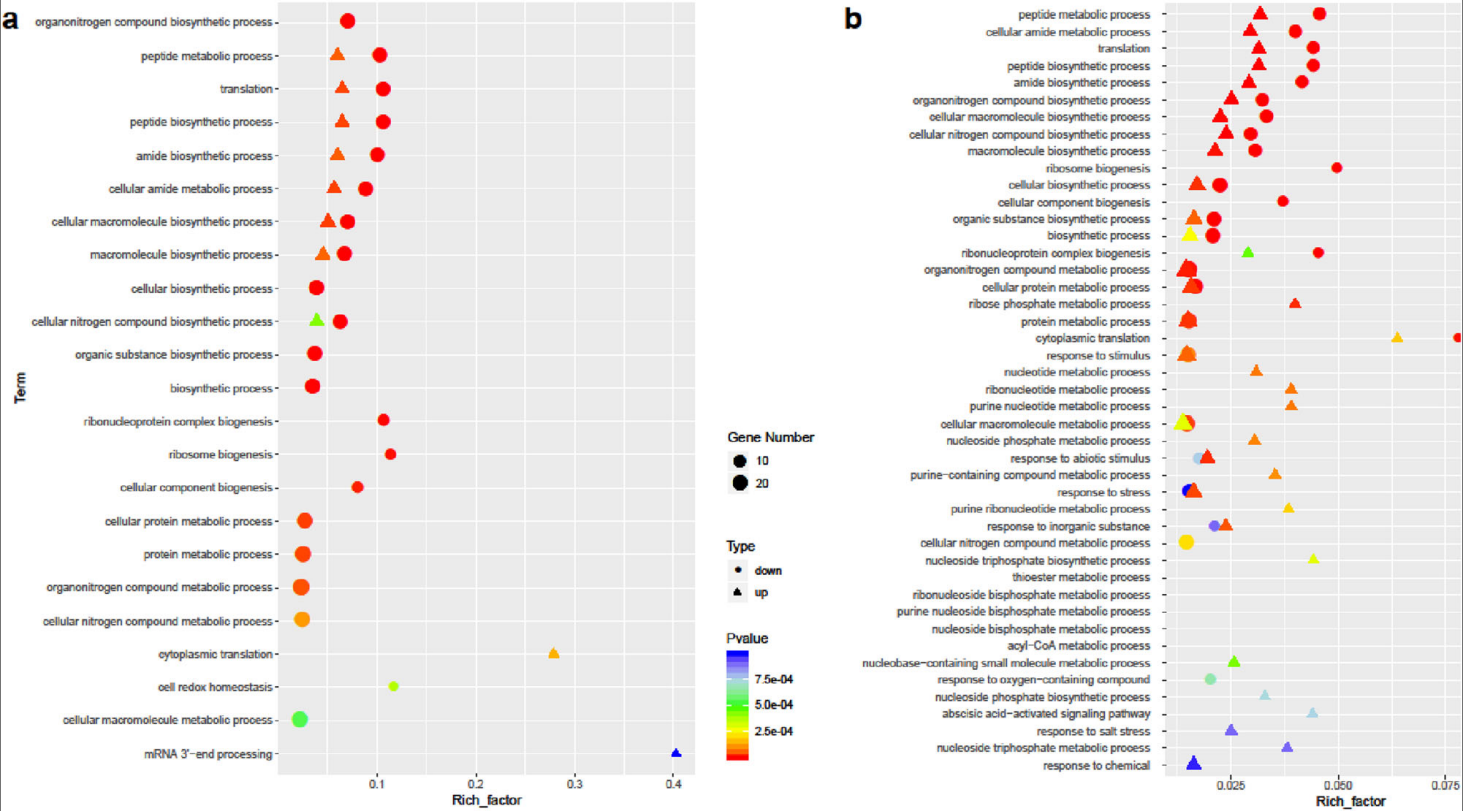
The enriched GO terms for mb-mRNAs in different conditions. **a** The enriched GO terms for mb-mRNAs at 24 °C; **b** the enriched GO terms for mb-mRNAs at 6 °C. The filled circles represent the GO terms for downwardly mb-mRNAs, and the filled triangles represent those for upwardly mb-mRNAs.

Relatively few mb-mRNAs were detected in heterografts grown at room temperature. To functionally characterize the biological roles of these mb-mRNAs, GO enrichment analyses were performed. The enriched GO terms were predominantly related to basic cellular activities and metabolism, such as “peptide biosynthetic process”, “translation”, “cellular macromolecule biosynthetic process”, and “cellular amide metabolic process”, regardless of the direction of movement (Fig. 6a; Table S5). This result suggests that mb-mRNAs are involved in basic growth and development of the scion and rootstock.

In chilling-stress conditions, three LATE EMBRYOGENESIS ABUNDANT (LEA) genes (*Cla020806*, *Cla021833*, and *Cla011408*), a sugar transporter gene (*Cla015944*) and a fatty-acid desaturase gene (*Cla013862*) were included among the downwardly mb-mRNAs. LEA proteins are well-known regulators of cold tolerance in plants^15–18^. Osmotic adjustment through sugar metabolism is also a well-known protective mechanism in plant cells^19,20^. The fatty-acid metabolite gene *FAD2* in *Arabidopsis* has been associated with the sensing of cold signals and maintenance of membrane viscosity homeostasis^21^. GO classification showed that GO terms such as “response to stimulus”, “response to stress” and “response to abiotic stimulus” were enriched in addition to GO terms related to basic life activities. Among the upwardly mb-mRNAs (upward-6 °C), the enriched GO terms were related to energy metabolism, such as “acyl-CoA metabolic process”, “nucleoside triphosphate metabolic process”, and “purine ribonucleotide metabolic process” (Fig. 6b; Table S5). The enrichment of the GO term “abscisic acid-activated signaling pathway” was also noted, as previous studies have revealed the involvement of ABA signaling in cold responses^22,23^. The abovementioned unique functional categorizations of both the downwardly and upwardly mb-mRNA under stress conditions are intriguing, as they would indicate different but orchestrated roles of the rootstock and scion in coordinating regulation of chilling responses (see Discussion below).

## Discussion

Large-scale investigations of RNA-Seq data have shed light on mb-mRNAs in various heterografting systems^10–14^. In recognition of the key technical constraints in mb-mRNA detection from previous studies, we propose an improved analytical pipeline to address them. First, we argue that whenever the genotypes of scions/rootstocks are different from the published reference genotypes, genome resequencing of the scion/rootstock should be performed to enable reconstruction of custom reference genomes. It is clearly demonstrated in our study that tens of thousands of SNPs and indels exist between the experimental and reference genomes, which, if not taken into consideration, would severely confound the detection of real mb-mRNAs. We showed that when directly using the published 97,103 and HZ gourd genomes for mb-mRNA detection, the number of mapped reads for each alignment was significantly reduced (at least 2 M less) (Table S6), which would become an important source of incorrect results.

Second, in searching for mb-mRNAs in heterografts, the homografted scion and rootstock should always be included to control for false positives. Although this caveat sounds intuitive^12,13^, here, we provide a quantitative analysis of how many false positives can arise during this step. We further verified these false positives by qRT-PCR (Fig. S1). False positives can also originate from unexpressed genes, which were taken into consideration in our pipeline as well. Intrinsic errors introduced by next-generation sequencing and the existence of gene families and homologous sequences in the plant genome are considered to be the main causes of many unintended predictions of mb-mRNAs.

Third, the read number cutoff for declaring an mb-mRNA matters. Since the identification of mb-mRNAs technically relies on the detection of scion- or rootstock-specific reads, the read count has a major impact on the results. In previous studies, read numbers from 1 to 4 were used as a threshold to declare mb-mRNAs^10–13,24^. In this study, we adopt FPKM (fragments per kb of transcript per million fragments mapped), a common measurement of transcript abundance in RNA-Seq analysis, to account for both gene length and sequencing depth^25^. An FPKM > 0.05 that equals ~0.05 fragments per kilobase of exon model per million mapped fragments was adopted as the criterion for identifying mb-mRNAs. The relatively high prediction accuracy rate (88.2%), as demonstrated by qRT-PCR, suggests that our methodology is reasonable. Last, sequencing depth matters. In light of the low abundance of many mb-mRNAs, Notaguchi et al.^14^ used a deep sequencing depth (327 million reads) to identify 138 mRNAs from *Arabidopsis*/*N. benthamiana* heterografts. Here, we used sequencing depths (>400 million reads) over eight times deeper than those for regular RNA-Seq analysis to increase the likelihood of identifying low-abundance mb-mRNAs. Our results are in agreement with those of Notaguchi et al.^14^ that the majority of mb-mRNAs are at a low or very low abundance in tissues.

The driving force of mRNA long-distance movement, i.e., whether an mRNA is diffused owing to high concentration or by active transport, is a basic biological question. Calderwood et al.^26^ postulated that mRNAs in companion cells with higher abundances are more prone to move. However, two recent studies failed to observe increased mobility of mRNAs when they were overexpressed^12,27^. Our study showed no apparent association between the abundance of mb-mRNAs and mRNA mobility and thus does not support the passive diffusion mode. Our observation that most of the mb-mRNAs originated from low-expressed genes further suggests the existence of active mechanisms that facilitate long-distance mRNA mobilization.

Based on the findings that mRNAs functioning in cold sensing, membrane homeostasis maintenance, and osmotic adjustment were transmitted downward while those involved in energy metabolism and ABA signaling pathways were transmitted upward, we propose that the scion may be the main site for chilling stress perception and convey the initial stress signal to the rootstock partly by mRNA downward transmission. This signal may subsequently trigger energy metabolism reprogramming and ABA-mediated stress responses in the rootstock, and by upward movement of effective mRNAs, it may enhance the stress tolerance of the scion. This conceptual simplified model, though requiring more solid experimental evidence, highlights an ordered and coordinated mb-mRNA network that includes signal interaction and feedback between the scion and the rootstock, underpinning heterografting advantages in plants under environmental stresses.

## Materials and methods

### Plant materials and treatments

The watermelon variety “8424” and the bottle gourd variety “YZ” were used as the scion and rootstock for heterografting, respectively. 8424 is a commercial cultivar sensitive to chilling; YZ is a rootstock variety showing excellent tolerance to low temperature. Homo- and heterografts between 8424 and YZ were generated following the cut-grafting method^9^. The grafted seedlings were placed in a growth chamber at 28 °C/22 °C (16 h/8 h) day/night temperatures with a relative humidity of 75%. After 15 days of recovery and growth, healthy, and uniform grafts were divided into two groups for room temperature (24 °C) or chilling treatment (6 °C). After 2 days of treatment, the leaves from the scion or rootstock of each grafted plant were sampled for RNA-Seq (Table S7). Two biological replicates were used for each graft combination.

REC values of each graft combination were measured using previously published protocols, with modifications^28^. In brief, 0.1 g of leaf tissue was immersed in 10 ml of deionized water for 6 h. The initial conductivity of the extraction solution (R1) was measured with a conductivity meter (Leici-DDS-307A, Shanghai, China). Then, the leaf tissue was boiled in deionized water for 20 min After cooling to room temperature, the conductivity of the extraction solution was measured again as the final conductivity (R2). The REC value was calculated as (R1/R2) × 100%.

### DNA extraction, genome resequencing, and reference genome modification

Genomic DNA was extracted from 8424 and YZ using the CTAB method^29^. Following quality verification by electrophoresis on a 1% agarose gel, the DNA samples were quantified to an equal concentration using a Qubit 3.0 (Fluorometer, Life Technologies, USA). One microgram of each DNA sample was used to construct paired-end DNA sequencing libraries using a TruSeq DNA Sample Prep Kit (Illumina, CA, USA). The Illumina HiSeq X sequencing platform was used for DNA sequencing.

To reconstruct the modified reference genomes for 8424 and YZ, the publicly available reference genomes of the watermelon variety “97,103”^30^ (Ref1) and the bottle gourd variety “HZ gourd”^31^ (Ref2) were used as the initial references. Resequencing data from 8424 and YZ were first mapped against Ref1 and Ref2, respectively, by using BWA (“aln” module, -e -1 -k 2). Alignment files were converted to BAM format files using SAMtools (v1.9), and then, initial SNPs and indels were identified by the SAMtools *mpileup* pipeline. To maximize sequence variant calls and to minimize false positives, sequence variants were filtered using the following criteria: (1) ≥6 reads, (2) homozygous variant, and (3) SAMtools mpileup phred-like quality score >20. Filtered variants were then incorporated into the modified reference genomes (Ref1-M and Ref2-M hereafter) by custom PERL scripts. Given that each genotype of a particular species has a unique genome composition in addition to SNPs and SVs in comparison to other genotypes, the unmapped reads were de novo assembled (-d 1 -D 1 -F -K 23) using SOAPdenovo2 (r241) to construct additional scaffolds, which were concatenated with the aforementioned modified genome sequences for further analysis. To remove potential contaminants in the assembled sequences, such as those from chloroplasts, mitochondria, bacteria, and viruses, BLASTn (*e* value < 1*e*^−5^) was used to search against the NCBI NT database.

### Total RNA extraction, RNA-Seq library construction, and sequencing

Total RNA was extracted using TRIzol Reagent (Plant RNA Purification Reagent for plant tissue) according to the manufacturer’s instructions (Invitrogen, Carlsbad, CA). The quality and concentration of the total RNA were evaluated by electrophoresis on a 1% agarose gel and by using an Agilent 2100 Bioanalyzer (Agilent Technologies, Santa Rosa, USA). Only RNAs with an integrity score >7 were used for library construction. Five micrograms of total RNA from each sample was used to construct an RNA-Seq library according to the TruSeq RNA Sample Preparation Kit from Illumina (San Diego, CA). In brief, each RNA sample was isolated by magnetic oligo (dT) beads and fragmented randomly into small pieces. Double-stranded cDNA synthesis with random hexamer primers (Illumina, San Diego, CA) was performed by using a SuperScript double-stranded cDNA synthesis kit (Invitrogen, Carlsbad, CA). The synthesized cDNAs were then subjected to end repair, phosphorylation, “A” base addition and ligation with adaptors according to Illumina’s library construction protocol. Sixteen RNA-Seq libraries (Table S7) were constructed for RNA Sequencing on the Illumina HiSeq X platform at Biozeron Biotechnology Co., Ltd. (Shanghai, China). The raw reads were filtered and trimmed using SeqPrep (https://github.com/jstjohn/SeqPrep) and Sickle (https://github.com/najoshi/sickle) with default parameters. After filtering, the clean reads were aligned to the corresponding modified reference genomes using TopHat (v2.1.0). The accession number for the sequence data is PRJNA553072.

### Bioinformatical procedures for mobile mRNA detection

A flowchart of the bioinformatical procedures for mobile mRNA detection is shown in Fig. 2b. The paired-end RNA-Seq reads from the scion (8424/YZ and 8424/8424, sampled sections are underlined) and rootstock leaves (8424/YZ and YZ/YZ) were first mapped by Bowtie2 (sensitive-local mode: -D 15 -R 2 -N 0 -L 20 -i S, 1, 0.75) to Ref1-M and Ref2-M, respectively. The mapped reads were not considered to be transmitted from the grafting partners and thus were excluded from further analysis, except for being used to control false positives from unexpressed but mistakenly predicted mb-RNAs by Cufflink (data sets C and C′ in Fig. 2b; for example, a watermelon sequence not expressed in the scion but detected in the rootstock). The unmapped reads from each sample were then BLASTn (-max_target_seqs 3 -e value 1e-5 -outfmt 6 -num_threads 8) searched against both Ref1-M and Ref2-M. The 8424/8424 reads matching Ref2-M (data set B in Fig. 2b) and the YZ/YZ reads matching Ref1-M (data set B′ in Fig. 2b) were also considered false positives. Therefore, only 8424/YZ reads matching Ref2-M (data set A in Fig. 2b) after subtracting false positives (data sets B + C′) were regarded as upwardly mb-mRNAs. Likewise, only 8424/YZ reads matching Ref1-M (A′ in Fig. 2b) after excluding YZ/YZ reads matching Ref1-M (B′ in Fig. 2b) and unexpressed genes (C in Fig. 2b) were regarded as downwardly mb-mRNAs (Fig. 2b). For stringency, only mb-mRNAs detected in both biological replicates were retained. We have deposited all related scripts in GitHub (https://github.com/orctyr/GraftRNAseq).

### Validation of mb-mRNAs

Quantitative real-time PCR (qRT-PCR) was used to validate the results of the in silico prediction of mb-mRNAs. To enable amplification of scion- or rootstock-specific sequences, the primers were always designed based on unique sequences between the two parts. cDNAs from 8424/YZ, YZ/YZ (positive control) and 8424/8424 (negative control) were amplified. Successful amplification of the positive control but no or very week amplification of the negative control indicated successful primer design. The ratio of expression level in the heterograft to that in the negative control was used as a quantitative measure to determine the authenticity of a predicted mb-mRNA.

For qRT-PCR, each extracted RNA sample was reverse transcribed using PrimeScript I (TaKaRa) following the manufacturer’s protocol. qRT-PCR was then carried out on an ABI prism 7900 Real-Time PCR System using a SYBR Premix ExTaq Kit (TaKaRa). The PCR program was as follows: initial polymerase activation for 30 s at 95 °C, followed by 40 cycles of 95 °C for 5 s and 60 °C for 30 s. The 2^–ΔΔCT^ method was used to normalize the raw qRT-PCR results of selected genes^30^. The bottle gourd *TuB-α* (tubulin alpha chain-like) gene (*BG_GLEAN_10019523*) was used as the reference gene. Primer sequences of the reference and target genes are listed in Table S8. Three replicates of each reaction were performed.

### Gene ontology (GO) enrichment analyses

Gene functions of the mobile mRNAs were retrieved from the watermelon and bottle gourd genome databases^31^^,32^. Gene functional categories (GO enrichments) were analyzed using Gorilla^33^ under a statistical significance threshold of *P* *=* 0.01. Prior to running the program, the mobile mRNA sequences were BLASTx searched against the *Arabidopsis* protein sequence database (https://www.arabidopsis.org/) under a statistical significance threshold of *P* *=* 0.01 to acquire legible sequence IDs for recognition in the program.

## Supplementary information

Fig. S1

Table S1–S2

Table S3

Table S4

Table S5

Table S6

Table S7–S8
