# Correction to: A universal pipeline for mobile mRNA detection and insights into heterografting advantages under chilling stress

**DOI:** 10.1038/s41438-020-0313-5

**Published:** 2020-05-06

**Authors:** Ying Wang, Lingping Wang, Nailin Xing, Xiaohua Wu, Xinyi Wu, Baogen Wang, Zhongfu Lu, Pei Xu, Ye Tao, Guojing Li, Yuhong Wang

**Affiliations:** 1 Institute of Vegetables, Zhejiang Academy of Agricultural Sciences, Hangzhou, 310021 China; 2 Institute of Vegetables, Ningbo Academy of Agricultural Sciences, Ningbo, 315040 China; 3 State Key Laboratory for Quality and Safety of Agroproducts, Zhejiang Academy of Agricultural Sciences, Hangzhou, 310021 China; 4 Biozeron Biotechnology Co., Ltd, Shanghai, 201800 China; 5 Present Address: College of Life Sciences, China Jiliang University, Hangzhou, 310018 China

**Keywords:** Abiotic, Bioinformatics

Correction to: *Horticulture Research*

10.1038/s41438-019-0236-1 published online 01 February 2020

After the publication of this article^1^, the authors became aware that there was an error in Fig. 2b that “Ref2-M” (below “Bowtie2”) on the right panel was mistakenly written as “Ref1-M”. The correct version is shown below.

**Figure 1:**
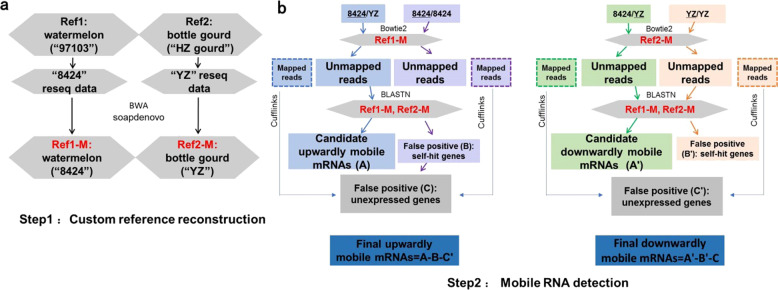
Fig. 2

The authors would like to apologize for this error.
