# Supplementary material for: A universal pipeline for mobile mRNA detection and insights into heterografting advantages under chilling stress
Source: Hortic Res. 2020 Feb 1;7:13. doi: 10.1038/s41438-019-0236-1 (PMC6994652; doi:10.1038/s41438-019-0236-1)
Supplement: Supplementary file 1 — Fig. S1 [file 41438_2019_236_MOESM1_ESM.doc]

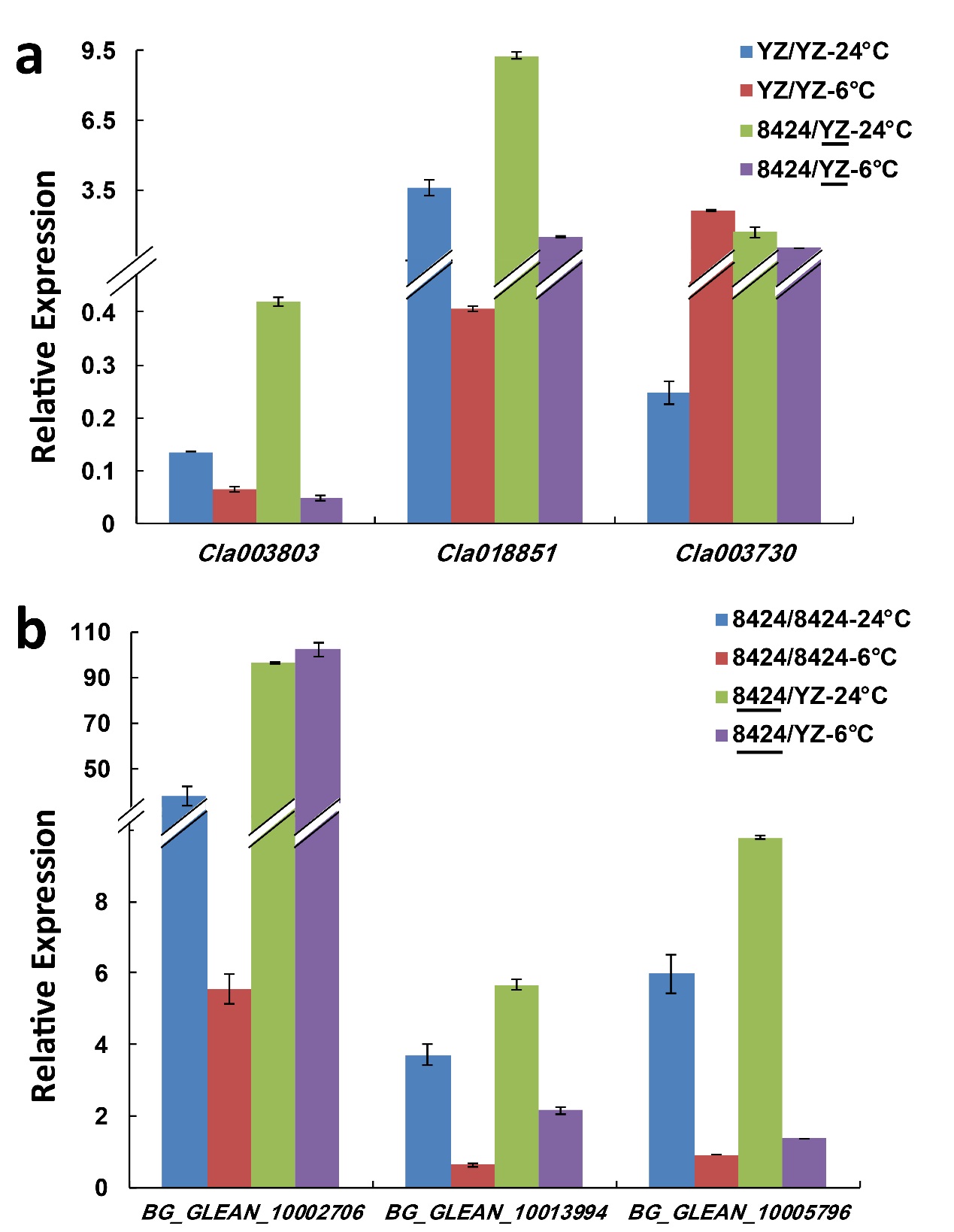


**Fig. S1. qRT-PCR validation of the false****positives in heterografts. a** Relative expression of downward mb-mRNAs. Because the watermelon mRNAs (*Cla003803*, *Cla018851*, *Cla003730*) were also detected in the bottle gourd, it indicated that these mRNAs were false positive mb-mRNAs; **b** relative expression of upward mb-mRNAs. Because the bottle gourd mRNAs (*BG_GLEAN_10002706*, *BG_GLEAN_10013994*, *BG_GLEAN_10005796*) were also detected in the watermelon, it indicated that these mRNAs were false positive mb-mRNAs.
