# Supplementary material for: A universal pipeline for mobile mRNA detection and insights into heterografting advantages under chilling stress
Source: Hortic Res. 2020 Feb 1;7:13. doi: 10.1038/s41438-019-0236-1 (PMC6994652; doi:10.1038/s41438-019-0236-1)
Supplement: Supplementary file 2 — Table S1–S2 [file 41438_2019_236_MOESM2_ESM.doc]

Table S1 Statistics of 8424 and YZ re-sequencing data

| Sample | Total reads | Total bases | Q20% | GC% | All mapped reads | Paired mapped reads |
| --- | --- | --- | --- | --- | --- | --- |
| 8424 | 64,417,420 | 9,480,437,064 | 98.68 | 35.76 | 48,993,447(88.68%) | 43,966,737(79.58%) |
| YZ | 74,566,000 | 10,867,660,630 | 98.22 | 35.45 | 53,121,651(82.64%) | 49,830,767(77.53%) |

Table S2 Statistics of assembled scaffolds in 8424 and YZ in addition to pseudo chromes

| Sample | Total scaffolds | Total bases  (bp) | GC% | Largest length(bp) | N50 scaffold | N50 length(bp) |
| --- | --- | --- | --- | --- | --- | --- |
| 8424 | 177,726 | 29,872,922 | 39.73 | 8,179 | 65 | 1,786 |
| YZ | 31,200 | 8,584,794 | 37.19 | 19,700 | 206 | 2,512 |
