# Supplementary material for: A universal pipeline for mobile mRNA detection and insights into heterografting advantages under chilling stress
Source: Hortic Res. 2020 Feb 1;7:13. doi: 10.1038/s41438-019-0236-1 (PMC6994652; doi:10.1038/s41438-019-0236-1)
Supplement: Supplementary file 7 — Table S7–S8 [file 41438_2019_236_MOESM7_ESM.doc]

Table S7 Sixteen RNA-Seq libraries used for RNA-Sequencing in this study

| Non-stressed (24°C) | Chilling-treatment (6°C) |
| --- | --- |
| 8424/8424-24°C-1 | 8424/8424-6°C-1 |
| 8424/8424-24°C-2 | 8424/8424-6°C-2 |
| YZ/YZ-24°C-1 | YZ/YZ -6°C-1 |
| YZ/YZ -24°C-2 | YZ/YZ -6°C-2 |
| 8424/YZ-24°C-1 | 8424/YZ -6°C-1 |
| 8424/YZ -24°C-2 | 8424/YZ -6°C-2 |
| 8424/YZ -24°C-1 | 8424/YZ -6°C-1 |
| 8424/YZ -24°C-2 | 8424/YZ -6°C-2 |

Table S8 Primer sequences used for qRT-PCR analyses (In parentheses are unsuccessful primers)

| Usage | Primer name | Sequence (5'-3') |
| --- | --- | --- |
|  | *BG_GLEAN_10019523*-F | AGCGTACCATTCAGTTTGTTGAT |
|  | *BG_GLEAN_10019523*-R | AGACCTCAGCAACACTGGTAGAG |
|  | *BG_GLEAN_10020833*-F | AAGTTTCTCTGATGGTATG |
|  | *BG_GLEAN_10020833*-R | ATAGCACTGAATAGGGAA |
|  | *BG_GLEAN_10000026*-F | TACAACCTTGGCAAGAACGA |
|  | *BG_GLEAN_10000026*-R | TACGCCACCCACAGAATTTA |
|  | *(BG_GLEAN_10003323)*-F | TCATCAGCTTCATCGCCTAC |
|  | *(BG_GLEAN_10003323)-*R | ACTGGGAACACACGAAACAA |
|  | *BG_GLEAN_10017931*-F | TCATATTGCTGCGGGTACAT |
|  | *BG_GLEAN_10017931*-R | AGTTGCTGACCCATACCACA |
|  | *BG_GLEAN_10027247*-F  *BG_GLEAN_10027247*-R | GCTCCGTGGCGGTATCA  AATGGTTGTGAGTTTACG |
|  | *BG_GLEAN_10015113*-F | AAGTTTCTCTGATGGTATG |
|  | *BG_GLEAN_10015113*-R | ATAGCACTGAATAGGGAA |
|  | *BG_GLEAN_10014162*-F | TACAACCTTGGCAAGAACGA |
|  | *BG_GLEAN_10014162*-R | TACGCCACCCACAGAATTTA |
|  | *(BG_GLEAN_10007517)*-F | TCATCAGCTTCATCGCCTAC |
|  | *(BG_GLEAN_10007517)-*R | ACTGGGAACACACGAAACAA |
|  | *BG_GLEAN_10015621*-F | TTGGTATTGCTACCGCACAT |
|  | *BG_GLEAN_10015621*-R | CCGGACGTCCATAGAAAGAT |
| Verification of mb-mRNAs | *BG_GLEAN_10006154*-F | GTTGAAAGGGATAAGTTGA |
|  | *BG_GLEAN_10006154*-R | AATGGTTGTGAGTTTACG |
|  | *Cla005931*-F | AAGTTTCTCTGATGGTATG |
|  | *Cla005931*-R | ATAGCACTGAATAGGGAA |
|  | *(Cla021880)*-F | TCATCAGCTTCATCGCCTAC |
|  | *(Cla021880)*-R | ACTGGGAACACACGAAACAA |
|  | *Cla008560*-F | TACAACCTTGGCAAGAACGA |
|  | *Cla008560*-R | TACGCCACCCACAGAATTTA |
|  | *Cla006530*-F | TCATATTGCTGCGGGTACAT |
|  | *Cla006530*-R | AGTTGCTGACCCATACCACA |
|  | *(Cla016275)*-F | GTTGAAAGGGATAAGTTGA |
|  | *(Cla016275)*-R | AATGGTTGTGAGTTTACG |
|  | *Cla005582*-F | AATGGTTGTGAGTTTACG |
|  | *Cla005582*-R | GCTCCGTGGCGGTATCAT |
|  | *Cla015757*-F  *Cla015757*-R  *(Cla018741)*-F  *(Cla018741)*-R | CGGCAGTTGACAGCCCTAG  CGGCAGTTGACAGCCCTAG  CGGCAGTTGACAGCCCTAG  CGGCAGTTGACAGCCCTAG |
|  | *Cla016341*-F | TACGCCACCCACAGAATTTA |
|  | *Cla016341*-R | TCATCAGCTTCATCGCCTAC |
|  | *BG_GLEAN_10013994*-F | ACACATGTCGCATAGAAGCC |
|  | *BG_GLEAN_10013994*-R | AAAGGGATCCTCCCAAAGGG |
|  | *BG_GLEAN_10002706*-F | TTGGGTCCTTAACCCAAGCA |
|  | *BG_GLEAN_10002706*-R | TCCCACCCTCCAAACACAAT |
|  | *BG_GLEAN_10005796*-F | CCGCCATGTTCATCCTTTGT |
| Verification of the | *BG_GLEAN_10005796*-R | CGGAGCCAGAGATGAGAACT |
| false positives | *Cla003803*-F | TGTAACTTGGGTGCTTGCTG |
|  | *Cla003803*-R | CAGGCCAGGGAGATAAACCA |
|  | *Cla018851*-F | CTTGAACAGACCCAGGTTCC |
|  | *Cla018851*-R | CCGCATAGATACCAGCTCCA |
|  | *Cla003730*-F | CCATTTGCCTTGGTTGCCTT |
|  | *Cla003730*-R | CAAAGCACACGCAACACG |
